# Molecular Biological Effects of Weak Low-Frequency Magnetic Fields: Frequency–Amplitude Efficiency Windows and Possible Mechanisms

**DOI:** 10.3390/ijms241310989

**Published:** 2023-07-01

**Authors:** Viacheslav V. Krylov, Elena A. Osipova

**Affiliations:** Papanin Institute for Biology of Inland Waters, Russian Academy of Sciences, Borok 152742, Russia

**Keywords:** low-frequency magnetic fields, ion parametric resonance, calcium, radical pairs, cryptochrome, mitochondria, biochemical oscillations

## Abstract

This review covers the phenomenon of resonance-like responses of biological systems to low-frequency magnetic fields (LFMF). The historical development of this branch of magnetobiology, including the most notable biophysical models that explain the resonance-like responses of biological systems to LFMF with a specific frequency and amplitude, is given. Two groups can be distinguished among these models: one considers ion-cofactors of proteins as the primary targets for the LFMF influence, and the other regards the magnetic moments of particles in biomolecules. Attention is paid to the dependence of resonance-like LFMF effects on the cell type. A radical-pair mechanism of the magnetic field’s influence on biochemical processes is described with the example of cryptochrome. Conditions for this mechanism’s applicability to explain the biological effects of LFMF are given. A model of the influence of LFMF on radical pairs in biochemical oscillators, which can explain the frequency–amplitude efficiency windows of LFMF, is proposed.

## 1. Introduction

Magnetic fields are constantly present in the environment. The natural geomagnetic field and its fluctuations accompanied the evolution of life on Earth. The current strength of the geomagnetic field varies from the equator to the poles from approximately 25 to 65 μT, and its direction varies in space in a rather complex way. The alternating constituent of natural magnetic fields is represented by low-frequency fluctuations, mainly due to regular and irregular processes in the Earth’s magnetosphere–ionosphere current system. The range of these magnetic fluctuations rarely exceeds hundreds of nT, and most natural variable magnetic fields are recorded in the range of up to 10 Hz [1]. Anthropogenic magnetic fields are superposed on the natural magnetic background due to the development of technology over the past century. These fields vary significantly in their characteristics [2]. The overall intensity of anthropogenic magnetic fields is steadily increasing year by year. In this regard, it is essential to understand the influence of the magnetic environment on organisms.

Weak (conditionally not higher than 100 μT) low-frequency magnetic fields (LFMF) are of great interest among the fluctuating magnetic fields. Their energy does not exceed the thermal noise, i.e., LFMF cannot directly affect an elementary act of chemical reactions of kT order [3]. At the same time, the biological effects of weak LFMF are often described in the literature [4]. Moreover, these effects can manifest themselves at specific values of the frequency and amplitude of the LFMF and be absent at other frequencies and amplitudes of the same order [5,6]. The researchers working in this field apply their efforts to clarify the mechanisms responsible for such biological responses. Over the last few decades, several hypotheses have been proposed in this regard. However, there is no definitive understanding of the formation of a specific biological response to LFMF.

This review describes resonance-like responses of biological systems to LFMF, presents the most notable biophysical models that tried to explain these effects in the past, and provides some future prospects.

## 2. Resonance-like Biological Responses to LFMF

**Figure 1:**
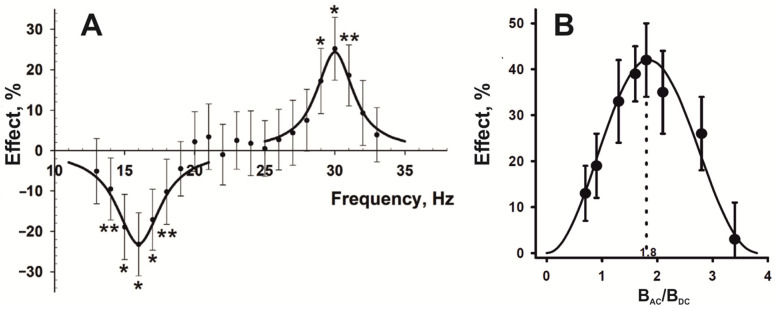
Examples of resonance-like biological responses to LFMF. (**A**) Dependence of regeneration rate in the planarian (*Schmidtea mediterranea*) on the frequency of LFMF. *B_DC_* = 40 µT; *B_AC_* = 74 µT. The approximating curves are shown as solid lines. The experimental data are shown as dots with average values and confidence intervals of 95%. * *p* < 0.001; ** *p* < 0.01. (**B**) The dependence of gravitropic reaction in segments of flax stems (*Linum bienne*) on the amplitude of FLMF. *B_DC_* = 46.5 µT; *f_AC_* = 35.8 Hz; *B_AC_*/*B_DC_* = 0.7, 0.9, 1.3, 1.6, 1.8, 2.1, 2.8, and 3.4. The approximating curve is shown as a solid line. The experimental data are shown as dots with the average values and standard errors. Adapted with permission from [5] 2022, Elsevier and [6] 2000, Maik Nauka/Interperiodica Publishing.

The first mentions of the resonance-like nature of biological effects on LFMF appear in the 1980s [7,8,9,10]. Subsequently, plenty of experiments were carried out on various biological test systems, wherein a non-linear relationship was observed between LFMP parameters and the magnitude of effects [5,6,11,12,13,14,15,16,17,18,19,20,21,22]. Moreover, the biological effects depend not only on the frequency (Figure 1A) [5,6,10,23] but also on the amplitude of an alternating magnetic field, *B_AC_* (Figure 1B) [6,7,10,11,12]. In other words, the researchers could observe the attenuation of effects with an increase in the frequency and-or amplitude of the applied LFMF. It implies specific primary targets for the LFMF influence in biosystems. Since resonance-like biological effects were revealed, scientists have been trying to explain the mechanisms of their occurrence.

## 3. Early Biophysical Models of Resonance-like Biological Effects of LFMF

### 3.1. Ions as Primary Targets

One of the first attempts to explain the dependence of biological effects on the frequency of LFMF was made by Abraham Liboff. He proposed the rather bold hypothesis of cyclotron resonance in living cells [24]. According to this hypothesis, the conditions necessary for the cyclotron motion of ions in the geomagnetic field exist in the transmembrane channels. This transmembrane transport changes when an alternating field with a cyclotron frequency (*f*_c_) calculated for biologically essential ions (calcium, potassium, magnesium, etc.) parallel to the static (most often the geomagnetic) field is added. It leads to biological effects [24]. The cyclotron frequency is calculated as

(1) $$f_{c}=\frac{1}{2\pi }\frac{q}{m}B_{DC}$$

where (*q/m*) is the charge-to-mass ratio of an ion and *B_DC_* is the static magnetic (geomagnetic) flux density. The “resonant” frequencies lie in the range from tens to hundreds of hertz for most biologically essential ions in the geomagnetic field.

Liboff’s model was criticized and rejected due to the impossibility of cyclotron motion in cells [25,26]. Despite this, testing the alternating fields with a cyclotron frequency for biologically essential ions led to robust and reproducible effects in different organisms. With the accumulation of empirical data, other features in biological responses have been revealed. It soon became clear that the magnetobiological effects depend not only on the frequency (Figure 1A), but also on the amplitude of an alternating magnetic field, *B_AC_* (Figure 1B) [7,10,11,12]. Moreover, the results obtained by different research groups were divided into two sets. On the one hand, experiments carried out by Blackman’s group to measure the nerve growth factor-stimulated neurite outgrowth in PC-12 cells, which indicated the maximum biological effect caused by the combination of resonant alternating and static magnetic fields with an intensity ratio of approximately *B_AC_*/*B_DC_*~0.9 [13]. Combined magnetic fields with this intensity ratio also affected calcium efflux from plasma membrane vesicles isolated from *Spinacia oleracea* L. [14] and inhibited growth in HeLa, VH-10, and Saos-2-His-273 cell cultures [15]. On the other hand, biological effects were found in alternating magnetic fields with a cyclotron frequency for various ions and with an amplitude exceeding the intensity of a static magnetic field by about 1.8 times [5,11,12,16,17,27,28,29,30,31]. To explain the biological effects of alternating magnetic fields with a frequency that formally corresponds to the cyclotron frequency for different ions and an amplitude ratio of *B_AC_*/*B_DC_*~0.9, Blackman and Blanchard proposed a model of ion parametric resonance [32]. At about the same time, Lednev published a model of parametric resonance in biosystems to explain the effects of fields with a *B_AC_*/*B_DC_* ratio of 1.8 [33,34]. Both biophysical models considered ions located in the ion-binding center of enzymes with ion-dependent activity as primary targets. It was assumed that an ion in the ion-binding center behaves as an oscillator in the static magnetic field. When applying an alternating field with parameters “tuned to the ion oscillations”, changes in the ion-dependent functioning of the enzyme can occur. It leads to biological effects [32,34]. The main difference between the models was the description of the dependence of the biological response appearance on the magnitude of the static and alternating magnetic fields by the Bessel functions *J*_1_(2*B_AC_*/*B_DC_*) and *J*_1_^2^(*B_AC_*/*B_DC_*) following Blackman and Lednev, respectively. The first function effectively approximated the experimental data for biological responses with maximum effects close to *B_AC_*/*B_DC_*~0.9, and the second to *B_AC_*/*B_DC_*~1.8. Disputes about the “correctness” of only one of the models were quite active. However, biophysicists overlook the following crucial detail.

The number of experimental confirmations of Blackman’s model in the literature is inferior to publications confirming Lednev’s model. It is noteworthy that the effects of alternating magnetic fields with a “resonance” frequency for different ions and *B_AC_*/*B_DC_* ratio of 0.9 are mainly described at the cellular and subcellular levels of organization [13,14,15,18,19]. Most often, specialized cell lines isolated from tumors were exposed, including the PC-12 cell line derived from a pheochromocytoma of the rat adrenal medulla [13,18,19] or the HeLa cell line derived from cervical cancer cells and the Saos-2-His-273 cell line derived from the primary osteosarcoma [15]. The molecular processes in such cells differ significantly from normal ones. In the case of alternating magnetic fields with a formal cyclotron frequency for biologically essential ions and the ratio of the amplitude of the alternating and the strength of the constant fields *B_AC_*/*B_DC_*~1.8, the exposures were mainly whole organisms. Most often, generalized biological parameters such as gravitropic bending in plant seedlings, the rate of tissue regeneration in planarians, changes in production parameters in daphnia, the degree of analgesia in snails, etc., were evaluated [5,11,12,16,17,29,31]. Lednev’s and Blackman’s models were tested simultaneously within the framework of one experiment, with an assessment of snails’ analgesia after exposure to magnetic fields. Lednev’s model was also confirmed in this study [12]. Recent work by researchers from Houston also confirms the selectivity of the influence of LFMF on different cell lines. An alternating magnetic field of complex configuration with a frequency of 50–350 Hz, created by rotating neodymium permanent magnets, induces highly selective cell death of patient-derived glioblastoma cells while leaving normal tissue cells undamaged [35], which opens up prospects for the therapeutic use of LFMF.

Thus, the Lednev and Blackman models look very similar. They noticeably differ in the amplitude of the effective alternating magnetic field and the test objects on which the hypotheses were experimentally tested. The differences in the effective amplitude of LFMF, which causes described above biological effects, could possibly be due to the specifics of cell cultures and whole organisms as test systems with different levels of biological organization. If the real mechanism of the perception of LFMF depends on the structural or functional features of the biomolecular environment around the primary targets, then the distortions in the predictions of the biophysical model are natural in individual test systems. In other words, the divergence of biological responses to the action of LFMF tuned to the same cyclotron frequency but with a different ratio of alternating and constant magnetic field intensity (0.9 or 1.8) could be two biologically determined cases of one molecular mechanism. Moreover, considering the criticism of the models [36], we can say that this mechanism most likely differs significantly from the concepts of Lednev and Blackman.

### 3.2. Magnetic Moments as Primary Targets

Subsequently, the resonance hypotheses were supplemented by models that viewed the magnetic moments of particles as primary targets [37,38,39]. Lednev suggested that weak microtesla and nanotesla alternating magnetic fields can affect the spins of electrons or hydrogen nuclei in biological molecules [39,40]. There is a uniform precession of magnetic moments around the field axis with the Larmor frequency in a static magnetic field. It was suggested that a superposed alternating magnetic field parallel to the static one lead to frequency modulation of the electromagnetic field generated by a magnetic moment that results in a biochemical effect. According to the analysis of the experimental data [39,40], a biological effect size is dependent on the ratio γ × *B_AC_*/*f*, where γ is the value of the gyromagnetic ratio for a given magnetic moment; *B_AC_* and *f* are the amplitude and frequency of the alternating magnetic field, respectively. The dependence of the magnitude of registered biological effects on the ratio γ × *B_AC_*/*f* follows theoretical expectations at γ = 42.577 Hz/μT and γ = 14,000 Hz/μT that corresponds to the nuclear spin of hydrogen and magnetic moments of electrons, respectively [20,21,22]. The authors registered pronounced maximums at γ × *B_AC_*/*f* = 0.9 and 2.75 and minor maximums at γ × *B_AC_*/*f* = 4.5 and 6.1. There was no biological effect at γ × *B_AC_*/*f* = 1.8; 3.8; 5.3; 6.7 [34,35,36]. It should be noted that most experimental verifications for this model were obtained by the only research group from the Institute of Theoretical and Experimental Biophysics RAS, which is doubtful. In contrast to models considering ions in the ion-binding center of proteins, the electrons or hydrogen nuclei-related model do not assume that biological effects depend on the strength of a static magnetic field but are inapplicable in the absence of a static magnetic field [39,40].

Binhi suggested several models for the biological effects of LFMFs [37,41,42,43]. The latest model proposed by the author also considers the precession of magnetic moments in a static magnetic field [37,41]. However, Binhi suggests that the influence of “resonant” LFMF parallel to the static one led to periodic temporary slowing: the magnetic moment did not move for a relatively large proportion of the precession half-period, that is, it hovered in a certain angular position, and then quickly made a complete revolution, then rests again, etc. [37]. This biophysical model has a wider scope. It explains not only the biological effects of LFMF but also the effects of hypomagnetic conditions, in which the precession of all magnetic moments should stop. According to Binhi’s model, the maximal biological effect will be caused by LFMF with a frequency *f* = γ × *B_DC_*. In contrast to Lednev’s model for magnetic moments, the biological effects of LFMF with a “resonant” frequency here depend on the *B_AC_*/*B_DC_* ratio. Following Binhi’s model, the maximal negative effects should appear at *B_AC_*/*B_DC_*~1.8 [37], i.e., at the same ratio that was proposed by Lednev in his model of parametric resonance in biosystems [33,34].

An intriguing feature of resonance models is the opposite biological effects provoked by LFMF with different parameters (Figure 1A) [5,11,17,23]. The opposite effects of magnetic fields with resonance conditions for calcium and potassium ions were explained in Lednev’s model of parametric resonance as follows. It is believed that despite the low affinity of potassium ions for the calcium-binding sites of proteins, they can compete with calcium ions for specific calcium-binding sites in proteins due to the high intracellular concentration of potassium ions [44]. At the same time, potassium cannot activate the enzymatic activity of the corresponding calcium-dependent proteins. It is assumed that the exposure of an organism to LF-MP with resonance parameters for calcium or potassium ions is accompanied by a change in the affinity of these ions for calcium-binding centers and a shift in the equilibrium in competition for the occupation of these centers and consequently causes the inhibition or activation of the activity of calcium-dependent proteins [11]. Binhi explains the opposite biological effects of LFMF in a relatively narrow range of frequencies/amplitudes with the thermal relaxation time and the rate of dependent biophysical or biochemical events [37,41].

It is also important to say that the choice of LFMF parameters for exposure is usually not strictly associated with the biophysical models in most papers reporting the effect of LFMF on biological processes. The technogenic environment forces scientists to check magnetic fields with an industrial frequency of 50 or 60 Hz. The reference to the above or other lesser-known biophysical models usually appears in the discussion sections of their papers to explain the possible mechanisms of the obtained results. Fewer works aim at testing the models by tuning the parameters of alternating and static magnetic fields to resonance conditions. Among these papers, slightly more experimental evidence is provided for Lednev’s parametric resonance model for biologically essential ions.

### 3.3. Some Inconsistencies in Biophysical Models

Among the results of experiments, some data do not agree with the above biophysical models of the LFMF influence on biomolecules. For example, significant biological effects caused by alternating magnetic fields directed perpendicular to a static (geomagnetic) field have been described [45,46,47,48]. Garcia-Sancho et al. [47] have estimated the uptake of radioactive ^42^K in several cell types (red blood cells, thymocytes, Ehrlich ascites tumor cells, HL60 human leukemia cells, and U937 human leukemia cells) exposed to parallel and perpendicular configurations of the static magnetic field and LFMF with the cyclotron frequency for sodium, calcium, and potassium ions. Only U937 human leukemia cells responded to both parallel and perpendicular configurations with an increase in the uptake of ^42^K [47]. In another work, Blackman et al. tested various mutual orientations of the static magnetic field and LFMF when studying their resonance model [46]. Both perpendicular and parallel configurations of static and alternating magnetic fields cause neurite outgrowth responses. In addition, these responses differed and included the enhancement and inhibition of neurite outgrowth registered in perpendicular and parallel configurations correspondingly [46]. It was noteworthy that the significant effects of the perpendicular static magnetic field and LFMF were described in tumor cell lines. It emphasizes the dependence of LFMF effects on the cell type described earlier.

Moreover, the dependence of the effects on the presence of light was shown when studying the influence of LFMF with resonance parameters for calcium and potassium ions on the nociceptive response in the terrestrial snail *Cepaea nemoralis* [17]. Thermal response latencies of snails injected with the enkephalinase inhibitor SCH34826 and exposed to “calcium or potassium resonance-tuned” magnetic fields were significantly higher in the light compared to the control (sham exposure). At the same time, there was no significant difference in the responses between exposed and unexposed individuals in the dark [17]. Such a dependence on light is characteristic of the radical-pair mechanism of the magnetic influence on living systems. 

However, the results of some experiments are not consistent with the above hypotheses. In addition, the great interest in magnetobiology in 1980–2010 gave rise to low-level works, primarily in poor control of generated magnetic field parameters. Thematic magnetobiological journals published instructions for the correct magnetobiological research to fix this situation [49,50,51]. However, one needs to keep in mind that some published experimental data may be incorrect.

## 4. Radical Pair Magnetoreception and Its Application to the Effects of Low-Frequency Magnetic Fields

Another promising hypothesis considers singlet-triplet transitions in radical pairs as a primary target for the influence of magnetic fields of geomagnetic order. Research in this direction originates from the discovery of the light-dependent orientation of birds in the geomagnetic field in the 1980s [52]. More precisely, it was found that birds can use the geomagnetic field for choosing their migrational direction only under blue-green light with a wavelength of 424–565 nm. The birds were disoriented under the illumination of the experimental area with yellow or red light with a wavelength of 590–650 nm [53,54]. Accurate experiments with robins show that the threshold of light-dependent perception of the magnetic field in this species lies in the interval between the wavelengths of 561 and 568 nm [55].

The necessity of photons with energy above a certain threshold for bird orientation in the geomagnetic field prompted Ritz and co-authors [56] to consider the idea that the primary magnetodetection is associated with the effect of magnetic fields on the electron spin state in radical pairs [57]. According to Ritz and co-authors, such radical pairs occur in cryptochrome molecules in the bird retina [56]. Cryptochromes are a class of blue light-sensitive flavoproteins found in various tissues in many plants and animals [58]. Cryptochrome molecules are structurally similar to the bacterial enzyme photolyase involved in DNA repair processes [59]. Cryptochromes and photolyase utilize flavin adenine dinucleotide (FAD) as a light-sensitive chromophore [60]. After the absorption of a photon with sufficient energy, the FAD enters an excited state. An electron from the excited FAD is sequentially transferred between tryptophan residues [61]. This electron transport reaction produces a deprotonated last tryptophan residue in this chain and a reduced FADH. It was suggested that cryptochrome with reduced FADH is in its active (or signal) state and long-lived radical pairs are formed during the process described above [62]. The inclination and/or intensity of an external magnetic field should affect the electron spin state of such radical pairs [56], i.e., the ratio of cryptochrome molecules in the signal and non-signal states should change depending on the direction of a static magnetic field [63]. This mechanism is currently viewed as the most likely basis for the magnetic orientation in birds and other animals, since the inclination of the geomagnetic field is place-specific depending on the latitude and some features of the Earth’s crust.

A lot of evidence for the crucial role of cryptochromes in biological magnetoreception has been accumulated to date [61,64,65,66,67,68,69,70,71]. Experiments with transgenic *Drosophila melanogaster* showed that the knockout of cryptochrome genes leads to the disorder of behavioral responses to magnetic fields [72]. Moreover, the expression of human cryptochrome (*hCRY2*) instead of the knocked-out genes led to the restoration of magnetosensitivity [73]. The amount of available data leaves no doubt that cryptochromes are the key proteins in the evolutionarily formed mechanism of animal orientation in the geomagnetic field, the so-called “chemical magnetic compass”. At the same time, significant magnetic field effects in the isolated molecules in vitro have been only detected at fields more than twenty times exceeding the geomagnetic one [61,65,67]. Even the most likely candidate for the role of an avian magnetosensor, cryptochrome 4, responded in vitro to a magnetic field on the order of thousands of microtesla [67]. Responses to the fields of the geomagnetic order have been shown so far on a model molecular triad consisting of covalently linked carotenoid, porphyrin, and fullerene moieties [74]. Probably, there must be an evolutionarily formed mechanism for enhancing the magnetic sensitivity of cryptochromes in the cells responsible for magnetic perception. The molecular environment and conformational rearrangements during magnetic perception may be of importance. Several scientific groups are currently working on this problem. In addition, attempts are ongoing to find alternative radical pairs in cryptochromes sensitive to weak magnetic fields [75]. For example, the radical pair formation between flavin and a superoxide radical [76,77,78] or ascorbyl radical [79] has been suggested.

It should be said that cryptochromes are a special case, and radical pairs are constantly present in biomolecules and affect almost all vital biochemical processes [80,81,82]. It allows us to consider radical pairs as primary targets for the influence of alternating magnetic fields regardless of their application to the magnetic orientation of birds and other animals. The lifetime of a radical pair in the individual free radical events takes place in the nanosecond to microsecond time scale [83,84]. The period of LFMF oscillations that influence organisms is often more than 16.67 ms (for LFMF fields with a frequency of 60 Hz or less). Therefore, LFMF can be viewed as static during the lifetime of a radical pair [84]. In other words, each radical pair experiences a different “quasistatic” magnetic field whose intensity (*B_QS_*) depends on the phase of the LFMF (α), which barely changes during the lifetime of the pair, with α randomly distributed between 0 and π [83]:

*B_QS_* = *B_DC_* + *B_AC_* cos α (2)

**Figure 2:**
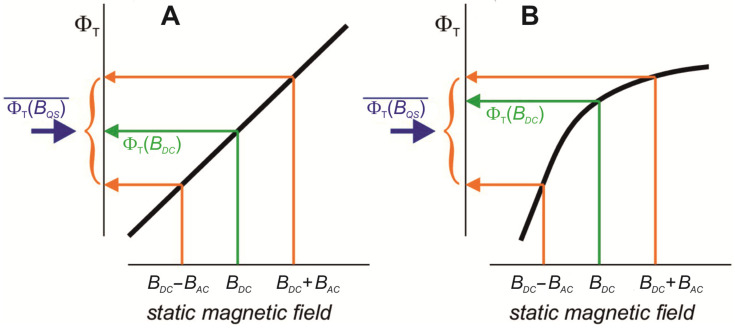
Schematic representations (thick black lines) of the dependence of the triplet reaction yield (Φ_T_) on the strength of a quasistatic magnetic field. The orange arrows indicate the yields for the maximum and minimum values of *B_QS_* in Equation (2) that correspond to extrema in sinusoidal LFMF. The green arrows show the yields when *B_QS_* = *B_DC_*. The blue arrows mark the free radical yields averaged over the phase of the LFMF. (**A**) When Φ_T_ depends linearly on *B*, the effect of the static magnetic (geomagnetic) field and the LFMF together equals that of the static field alone; the blue and green arrows are at the same point of the ordinate axis, i.e., there is no effect of LFMF. (**B**) When Φ_T_ depends non-linearly on *B*, the effects of static magnetic field plus LFMF and static magnetic field alone differ. Φ_T_ in (**B**) has been drawn with negative curvature (concave downward), with the result that the triplet reaction yield under LFMF plus the static magnetic field is lower than the static magnetic field alone. A positive curvature (concave upward) would give the opposite signs. Adapted with permission from [83] 2019, eLife Sciences Publications, Ltd.

Aspects of the possible impact of such a quasistatic LFMF on the radical pairs have been viewed by Peter Hore [83]. It is important that the dependence between singlet and triplet yields and the intensity of the applied magnetic field in the range of *B_DC_* ± amplitude of *B_AC_* must be non-linear for the LFMF to affect the course of a radical-pair reaction. In this case, LFMF can shift the equilibrium between singlet and triplet yields relative to the equilibrium state in a static geomagnetic field (Figure 2). Another essential concept is the so-called “weak field effect” that appears under magnetic fields of the order of tens and hundreds of microtesla. This effect has been shown in experiments with different radical pair reactions [61,85,86,87,88]. It provides the non-linearity of the dependence of the yield of a radical-pair reaction on the strength of a constant magnetic field [89]. That is, the probability of compliance with the “nonlinear dependence” condition is maximal in magnetic fields of the geomagnetic order.

The radical-pair mechanism can explain a significant part of the effects of low-frequency magnetic fields with an amplitude of tens to hundreds of microtesla. However, it cannot explain the effects of weaker fields. The simulation performed by Peter Hore [83] for a radical pair, [FAD^•−^ Z^•^] with a lifetime of 1 µs, under *B_DC_* = 50 µT and LFMF of 1 µT, 50 Hz, showed that the largest change in the reaction yield caused by the LFMF is −14 ppm parts per million (ppm). The radical pair [FAD^•−^ Z^•^] is identical to [FAD^•−^ TrpH^•+^] except that the second hypothetical radical, Z^•^, has no hyperfine interactions and is more sensitive to weak magnetic fields. For the [FAD^•−^ TrpH^•+^] radical pair, such a simulation resulted in −1.2 ppm [83]. These values seem small, and it is difficult to imagine that changes in the accumulation of singlet and triplet products of radical-pair products are directly related to the effects of LFMF with an induction of less than 1 μT. Although the radical-pair mechanism can clarify the dependence of biological effects on the amplitude of LFMF [7,10,11,12,13], it is hard to directly explain the frequency effectiveness windows of LFMF [5,10,11,40,90].

## 5. Future Prospects

### 5.1. Incomprehension of the Molecular Pathways Forming Magnetobiological Effects

One of the main challenges facing scientists today is to clarify the molecular pathways of transforming the perceived magnetic impact into registered biological effects. All resonance-like models were developed using experimental results at the cellular, organismal, and even population levels. A network of signaling pathways and molecular interactions between the putative primary targets and biological effects remain unknown. These pathways and the intracellular environment of magnetosensitive molecules can play a crucial role in the occurrence of magnetobiological effects. The same challenges face researchers investigating the influence of the geomagnetic field on radical-pair reactions in cryptochrome. Protein–protein interactions and conformational changes in molecules in the process of radical-pair magnetoreception can be the amplifier that allows the detection of minor changes in the geomagnetic field. For example, Qin et al. [91] reported that the protein encoded by the *CG8198* gene can bind to cryptochrome and promote its magnetoreceptor function. Further search for long-life radical pairs in biomolecules and pathways for signal transmission is required.

Probably, several different primary targets perceive LFMF in cells. This assumption complicates the interpretation of emerging biological responses. Therefore, magnetobiological experiments with test systems that evaluate target magneto-sensitive biochemical processes are required. Unlike biological parameters at the organismal level, such indicators registered “closer” to the primary targets will give a more unambiguous interpretation.

### 5.2. Oscillating Biochemical Processes as a Reason for Resonance-like Responses of Biological Systems to LFMF

The dependence of the manifestation of the effects of LFMF with “resonance parameters for calcium and potassium ions” on the presence of light [17] makes one think about the radical-pair nature of these resonance-like biological responses. However, the following question arises: how can the frequency–amplitude windows of LFMF biological efficiency be explained? A general hypothesis, which combines the resonance-like responses of biological systems to LFMF and the effects of magnetic fields on radical pairs, is given below.

1. Radical pairs are the main target for LFMF’s influence on organisms. If radical pairs emerge in biochemical processes that oscillate in cells with the frequency *f* _OSC_, and this emerging occurs in a specific phase of the oscillations, then the manifestation of biological effects can depend on the frequency of LFMF.
2. The altered or “signal” state of a given oscillating process depends on the ratio of the singlet and triplet yields of the radical-pair reaction included in it.
3. If an external LFMF with frequency *f* = *f* _OSC_ and amplitude *B_AC_* parallel to the static (geomagnetic) field (*B_DC_*) is applied to such an oscillating chemical process, then due to the negligible lifetime of radical pairs and depending on phase coincidence, some radical pairs of the biochemical oscillators will be located at the resulting magnetic field *B_DC_* + *B_AC_* throughout the whole LFMF exposure. The same part of the oscillators will generate radical pairs exposed to *B_DC_* − *B_AC_* throughout the whole LFMF exposure. Most of the radical pairs will be under a “quasistatic” magnetic field with the intensity from *B_DC_* − *B_AC_* to *B_DC_* + *B_AC_* throughout the exposure.
4. According to Hore [83], changes in the ratio of singlet and triplet yields of a biradical reaction in response to LFMF occur if there is non-linear dependence between the singlet–triplet reaction yields and the magnetic field strength within limits from *B_DC_* − *B_AC_* to *B_DC_* + *B_AC_* (Figure 2B). Synchronization of LFMF frequency with the frequency of chemical oscillations provides a quasistatic “effective” magnetic field for radical pairs in a portion of chemical oscillators. The ratio of triplet and singlet yields for this portion of oscillators will differ from the state for the rest of the oscillators throughout the whole LFMF exposure due to the non-linear dependence between the triplet and singlet yields and magnetic field intensity as the “low field effect”. A notable change in LFMF frequency (*f* ≠ *f* _OSC_) leads to a condition where radical pairs regularly generated by a chemical oscillator will experience quasistatic magnetic fields of different intensities at different moments. The disappearance of the biological effect at a changed non-resonant LFMF frequency can be a consequence of the inability to maintain a “signal” state of the portion of the biochemical oscillators throughout the LFMF exposure. It ensures the appearance of frequency windows of magnetobiological effects.
5. The “low field effect” [89] provides “non-linear dependence” conditions; therefore, the biologically effective amplitude of the LFMF exists for a specific radical-pair reaction. A change in this amplitude can shift the magnetic field intensity values to the area of linear dependence, which leads to the absence of a biological effect (Figure 2A). It explains the amplitude windows of the LFMF efficiency.

We can consider a specific example to show the viability of the above model describing amplitude–frequency effective windows in the responses of biological systems to LFMF within the framework of a radical-pair mechanism. Most experiments testing ion-related resonance models are performed with fields formally “tuned to the cyclotron frequency for the calcium ion” [5,9,10,11,16,29]. This frequency for calcium ions in the geomagnetic field at temperate latitudes of 40–55 µT will be about 30–42 Hz. These values have often been described as *B_DC_* and *f_AC_* in the experiments testing resonance biophysical models for calcium ions [5,6,16,29,30,90]. The 30–42 Hz frequency should correspond to a chemical oscillation period of approximately 24–33 ms. The biologically effective amplitude of the LFMF tuned to calcium ions provides a quasi-static magnetic field relative to the lifetime of radical pairs from approximately 0 to 0.1 mT (for *B_AC_*/*B_DC_*~0.9 in the case of Blackman’s model) or from roughly −0.05 mT through 0 up to +0.15 mT (for *B_AC_*/*B_DC_*~1.8 in the case of Lednev’s model).

**Figure 3:**
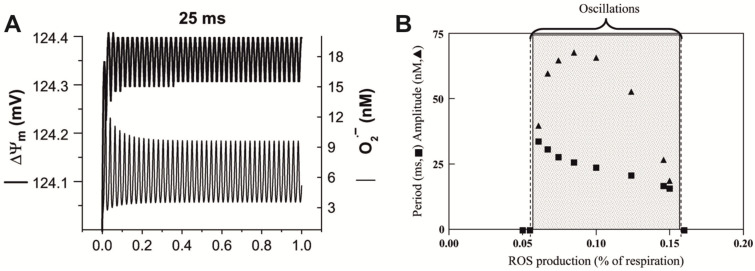
Frequency and amplitude modulation of the mitochondrial oscillator model through changes in the balance between reactive oxygen species production and scavenging. (**A**) An oscillation period of 25 ms is shown for superoxide dismutase concentrations of 0.75 μM. The model parameters are detailed in [92,93,95]. (**B**) Under similar parametric conditions, the frequency and amplitude of the oscillations in the superoxide anion delivered to the cytoplasm as a function of the fractional superoxide anion production (ms). Within the oscillatory region (shaded), the oscillatory period constantly decreased, whereas the amplitude reached a peak and then decreased as a function of the increase in reactive oxygen species production. Adapted with permission from [93] 2006, Elsevier.

The described effects [5,6,16,29,30,90] can be explained by the above model applied to radical pairs in chemical oscillators on the mitochondrial membrane [92]. Under unstressed “physiological” conditions, the mitochondrial membrane potential (ΔΨm) fluctuates with a period close to 25 ms (Figure 3A). This oscillation is associated with fluctuating levels of cytoplasmic superoxide anions in the nM range. Its period can vary from 34 ms to 16 ms depending on the balance between reactive oxygen species production and scavenging (Figure 3B). These oscillations become low-frequency and high-amplitude with the transition of mitochondria to the “pathophysiological” state [93]. Scilicet, oscillatory processes with the 30–42 Hz frequency that formally corresponds to the cyclotron resonance frequency for calcium ions in the geomagnetic field at temperate latitudes (40–55 µT) exist in mitochondria under normal conditions. The chemical composition of this oscillatory process includes radical-pair reactions as a shunt of electrons of the respiratory chain towards the generation of superoxide anions, following the transport and scavenging of superoxide radicals by superoxide dismutase [92]. For example, Complex II of the mitochondrial respiratory chain includes the oxidation of succinate by flavin in the substrate pocket. It is a two-electron process, and the passage of single electrons along the three [FeS] centers to the ubiquinone gives rise to three different biradical pairs that may be subject to the influence of an external magnetic field [94]. Two radicals involved in this process, flavin and quinone, could reduce paramagnetic O_2_ to a superoxide. If an external magnetic field can affect any of these redox electron pairs, then they may be trapped in a spin-forbidden state, inhibiting electron passage [94].

These and other radical-pair processes in mitochondria may be responsible for the biological effects of formally “calcium-tuned” fields with characteristic frequency–amplitude windows with the following two assumptions: (1) the ratio of triplet and singlet yields depend non-linearly on the magnetic field within 0–0.1 (or 0–0.15) mT; (2) the ratio of triplet and singlet yields at some stage of the oscillation affects the functional parameters of mitochondria. Moreover, changes in mitochondria functioning can explain most of the biological effects obtained in earlier experiments with formally “calcium-tuned” LFMF, such as changes in diatom mobility [10], neurite outgrowth [90], planarian regeneration [5,11,29], gravitropic reaction [6], etc. Moreover, the proposed mechanism probably underlies the biological effects of fields with higher frequencies if they are biochemical oscillators’ frequency harmonics. In this regard, the study of a possible relationship between the selective influence of LFMF on cancer cells, related to reactive oxygen species in the mitochondria [35,94] with fluctuations in the levels of cytoplasmic superoxide anion and mitochondrial membrane potential [92,93], is of great interest for application in oncology therapy.

This example explains the frequency–amplitude efficiency windows of LFMF with a frequency corresponding to Lednev’s or Blackman’s “calcium ions resonance” from the standpoint of the magnetic field effect on radical pairs in the 30–42 Hz biochemical oscillator in mitochondria. One can test the proposed assumptions since it is possible to register the period of mitochondrial oscillations, and the frequency of these oscillations can be changed with inhibitors [92,93]. The effective frequency of LFMF should change when the frequency of fast low-amplitude mitochondrial oscillations alters. If the hypothesis is supported, a further research direction could be the search for additional biochemical oscillators [96] that generate radical pairs. It probably could help to explain the biological effects of LFMF with other frequencies and amplitudes.

## Data Availability

No new data were created or analyzed in this study.
